# Venous Thromboembolism (VTE) in Post-Prostatectomy Patients: Systematic Review and Meta-Analysis

**DOI:** 10.3390/jcm12123979

**Published:** 2023-06-11

**Authors:** Mudassir Wani, Abdullah Al-Mitwalli, Subhabrata Mukherjee, Ghulam Nabi, Bhaskar K. Somani, Jayasimha Abbaraju, Sanjeev Madaan

**Affiliations:** 1 Department of Urology, Swansea Bay University Health Board, Swansea SA6 6NL, UK; mudassir.wani@nhs.net (M.W.); abdullah.al-mitwalli@wales.nhs.uk (A.A.-M.); 2 Department of Urology, Imperial College Healthcare NHS Trust, London W6 8RF, UK; subhabrata.mukherjee@nhs.net; 3 School of Medicine, Centre for Medical Engineering and Technology, University of Dundee, Dundee DD1 9SY, UK; g.nabi@dundee.ac.uk; 4 Urological Surgery Department, University Hospital Southampton NHS Foundation Trust, Southampton SO16 6YD, UK; b.k.somani@soton.ac.uk; 5 Department of Urology & Nephrology, Dartford, and Gravesham NHS Trust, Dartford DA2 8DA, UK; jayasimha.abbaraju@nhs.net

**Keywords:** prostate cancer, radical prostatectomy, venous thromboembolism, mechanical prophylaxis, pharmacological prophylaxis

## Abstract

Radical prostatectomy (RP) is one of the recommended treatments to achieve oncological outcomes in localized prostate cancer. However, a radical prostatectomy is a major abdominopelvic surgery. Venous thromboembolism (VTE) is a well-known complication associated with surgical procedures, including RP. There is a lack of consensus regarding VTE prophylaxis in urological procedures. The aim of this systematic review and meta-analysis was to investigate different aspects of VTE in post-radical prostatectomy patients. A comprehensive literature search was performed, and relevant data were extracted. The primary aim was to perform a systematic review and meta-analysis (wherever possible) of VTE occurrence in post-RP patients in relation to surgical approach, pelvic lymph node dissection, and type of prophylaxis (mechanical or combined prophylaxis). The secondary aim was to investigate the incidence and other risk factors of VTE in post-RP patients. A total of 16 studies were included for quantitative analysis. Statistical methods for analysis included the DerSimonian–Laird random effects. We were able to conclude that the overall incidence of VTE in post-radical prostatectomy is 1% (95% CI) and minimally invasive procedures (MIS), including laparoscopic, as well as robotic procedures for radical prostatectomy and RP without pelvic lymph node dissection (PLND), are associated with less risk of developing VTE. Additional pharmacological prophylaxis to mechanical methods may not be necessary in all cases and should be considered in high-risk patients only.

## 1. Introduction

Urological cancer surgical procedures have evolved remarkably over the years. The open surgical methods have now largely been replaced by endoscopic or minimally invasive procedures including laparoscopy and robot-assisted methods. Perioperative venous thromboembolism (VTE), including deep vein thrombosis (DVT) and pulmonary embolism (PE), represents a serious and potentially fatal complication after urological cancer surgeries [1,2]. DVT incidence, without prophylaxis, has been estimated to occur among 10–40% of medical and general surgical patients [3]. PE results in approximately 10% of hospital deaths—the most common cause of inpatient deaths [4]. Furthermore, VTE accounts for the most common preventable cause of hospital-related deaths [5,6,7]. National Surgical Quality Improvement Program (NSQIP) data revealed a 2.5-fold increased risk of VTE within 30 days in abdominal procedures compared to other procedures, such as breast surgery [8]. One of the most common abdominal urological cancer surgeries is radical prostatectomy (RP), performed for prostate cancer (PCa), which also evolved from open to robotic-assisted surgery. Ultrasound evaluation of VTE occurrence in post-RP patients is estimated at 20% [9].

Thromboprophylaxis helps in reducing morbidity and mortality in surgical patients, using either mechanical methods to promote venous outflow from the legs, or pharmacological prophylaxis (PP) in the form of antithrombotic drugs [10]. The efficacy of thromboprophylaxis in decreasing the incidence of venous thromboembolic events has been demonstrated in randomized controlled clinical trials [11]. Similarly, meta-analyses by various specialties looking into VTE prophylaxis in abdomen/pelvic surgeries concluded that anticoagulants, such as low molecular weight heparins (LMWH), decrease the relative risk of VTE by approximately 50%. However, LMWH administration also increases the relative risk of major bleeding by approximately 50% [12,13]. Regarding prophylaxis after RP, there is a lack of high-quality evidence or standard practice across the globe. In the UK, 98% of patients receive PP after RP (which we also observed in our study), while only 17.8% receive PP after RP in the USA [11,14]. One US study found that 30% of patients post-RP received no VTE prophylaxis [14].

The aim of this systematic review and meta-analysis was to investigate different aspects of VTE in post-RP patients. The primary aim was to perform a systematic review and meta-analysis (wherever possible) of overall VTE occurrence in post-RP patients, as well as VTE occurrence in relation to surgical approach, pelvic lymph node dissection, and type of prophylaxis (mechanical or combined prophylaxis). The secondary aim was to investigate the impact of other risk factors of VTE in post-RP patients.

## 2. Material and Methods

### 2.1. Protocol and Registration

This systematic review is registered with the PROSPERO International Registry (CRD42022364222).

### 2.2. Evidence Acquisition

The inclusion criteria for studies included the following:

1. Population: male patients, prostatectomy for prostate cancer;
2. Intervention: pharmacological/combined prophylaxis (PP) for VTE;
3. Comparator/control: no prophylaxis or mechanical prophylaxis for VTE.

The exclusion criteria for studies included the following:

1. Population: prostatectomy for non-prostate cancer or part of other surgery, such as cystoprostatectomy.
2. Intervention: if the interventions are ill-defined or structural methods are inadequate;
3. Comparator/control: studies that lacked proper grouping into control, and intervention;
4. Study design: studies that did not fulfill the above criterion and lacked any defined outcomes.

### 2.3. Outcome Measures

1. Primary outcomes: VTE occurrence with
  - Overall incidence of VTE in post-RP Patients;
  - Surgical approach: open, minimally invasive (laparoscopic or robot-assisted laparoscopic prostatectomy);
  - Pelvic lymph node dissection (PLND);
  - Prophylaxis (no prophylaxis, mechanical only, pharmacological only, combined).
2. Secondary outcomes:

Risk factors for VTE in post-RP patients include age, body mass index (BMI), smoking, and personal or family history of VTE. 

### 2.4. Search Methods

We followed the “Preferred Reporting Items for Systematic Reviews and Meta-analyses (PRISMA)-2020 protocol, for this systematic review [15]. The PRISMA Checklist for this review is shown in Supplementary Table S1. An electronic search was carried out into MEDLINE, EMBASE, and Cochrane Library with results from Cochrane CENTRAL, ClinicalTrials.gov, and the International Clinical Trials Registry Platform (ICTRP). There was no date or language limit. Search terms included ‘Prostatectomy’, ‘Radical’, ‘Cancer’, ‘Venous thromboembolism’, ‘Deep Vein Thrombosis’, ‘Pulmonary Embolism’, ‘Prophylaxis’, ‘Pharmacological’, ‘Mechanical’, and ‘surgical procedures.’ Boolean operators (‘And’/‘Or’) were used. The search strategy is attached as Supplementary Table S2. The search was performed on 21 July 2022.

### 2.5. Study Selection

Rayyan software (a free web tool designed to help researchers speed up the process of screening and selecting studies) was used to aid in the process of duplicate removal and initial screening, and to facilitate author collaboration. After deleting duplicates, following the inclusion and exclusion criterion, studies were shortlisted. Full-text papers of the shortlisted studies were reviewed by the SQ3R (Survey, Question, read, recite, review) technique. The process of reviewing studies was completed independently by three authors (M.W, A.M, S.Mu, J.A). In case of any disagreements, the discussion was carried out with the rest of the authors (S.Ma, G.N, B.S) to reach a consensus. 

### 2.6. Data Extraction

The data was initially divided into two parts. The first part consisted of a detailed description of all the studies included. The second part consisted of grouping data into columns—the number of patients, type of surgical approach procedure, pelvic lymph node dissection, post-op VTE prophylaxis (duration/type of medication), and additional VTE (mechanical). Data extraction was performed by two authors independently (M.W, A.M).

### 2.7. Quality Assessment

For quality assessment of the studies included, the Newcastle–Ottawa Scale (NOS) was used [16]. 

### 2.8. Statistical Analysis

The statistical analysis was carried out in three parts:

a Statistical evaluation of overall VTE occurrence.

The meta-analysis pooled together the results from the different studies, calculating the overall percentage of patients in which the outcomes occurred. Due to the rare occurrence of the outcomes, the Freeman–Tukey double arcsine transformation was performed before analysis. This was used to stabilize the variances when the proportions were close to zero, and a normal approximation to the binomial distribution did not hold.

The DerSimonian–Laird random-effects method was used for the analysis, regardless of the degree of heterogeneity between the study results. Heterogeneity between studies was assessed based on the significance of the between-study heterogeneity and on the size of the I^2^ value. Substantial heterogeneity was assumed if the I^2^ value was above 50%.

b Statistical evaluation of VTE occurrence depending on the type of surgical procedure (Open/Minimally Invasive Surgery (MIS)) and whether PLND was performed or not.

Meta-analysis methods were used to pool together the within-study differences in the outcome of different procedures. The outcome of interest was the occurrence of VTE, which was binary in nature. Differences between procedures were expressed as a relative risk. The DerSimonian–Laird random-effects method was used for the analysis as mentioned earlier.

Data was collected from a series of studies. However, not all studies collected data from more than one type of procedure, and thus a comparison between procedures was based on data from a more limited number of studies. The data collected enabled two different comparisons to be made:

- MIS procedures vs. open procedures;
- Procedures using PLND vs. procedures without PLND.

c Statistical Evaluation of VTE occurrence depending on the method of prophylaxis used (mechanical or combined).

The systematic review found only two studies that collected data on both methods and that could be used in a within-study comparison. However, additional studies collected data on either one method or the other. To be able to include all studies in the analysis, a within-study comparison was not performed. Instead, the occurrence of VTE was pooled separately for the mechanical and combined methods. The difference in VTE occurrence between methods was assessed based on the significance of the between-group heterogeneity. Similar statistical methods were used as previously described for overall VTE occurrence.

## 3. Results

### 3.1. Study Selection Results

**Figure 1:**
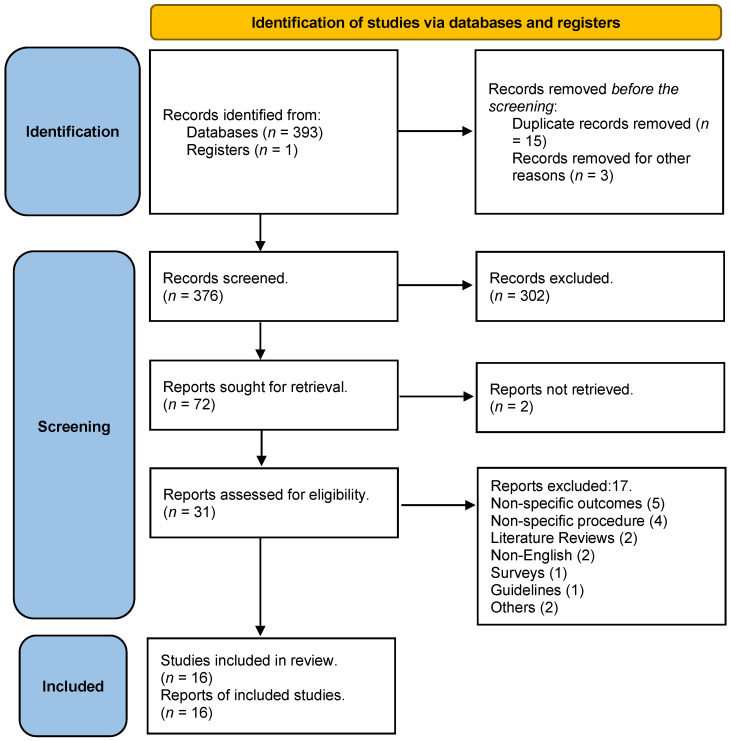
PRISMA 2020 Flow diagram.

In the initial search, 394 studies were identified. A total of 18 were removed (including 15 duplicates). In total, 376 studies were screened. After the title and abstract review, 302 studies were excluded. Full-text retrieval was sought for 74 studies; however, we could retrieve only 72. Out of 72 studies, 41 studies were excluded after a full-text review. The remaining 31studies were scrutinized for eligibility criteria, and in the end, 16 studies were included in the systematic review. The PRISMA-2020 flow diagram is shown in Figure 1.

### 3.2. Quality Assessment Results

The quality assessment results of the included studies are shown in Table 1.

### 3.3. Study Characteristics

All sixteen studies included are summarized in Table 2, highlighting the study type, period of study, main characteristics/methodology, and conclusions.

### 3.4. Clinical-Pathological Results

The clinical-pathological results are summarized in Supplementary Table S3. The main highlight is that surgery was usually performed for T2-stage prostate cancer (54%).

### 3.5. Demographics and VTE Risk Factors

The VTE risk factors including age, BMI, personal history of VTE, as well as family VTE, are summarized with remarks in Table 3. The age was described in 13 studies, with the mean age of patients undergoing surgery being 64 years (60–72 years). Six studies provided details about BMI. The average BMI was 27.6 kg/m^2^. Personal history of VTE had been investigated by three studies, with the average being 2.3% and family history of VTE in one study, 4.0%. Three studies investigated smoking and were found in 30% of patients. Lastly, only one study investigated Caprini’s score in their cohort of patients.

### 3.6. Surgical Procedure Results

Out of 16 studies, 15 have mentioned detailed surgical approaches offered for radical prostatectomy (Open/Laparoscopic/Robotic). In total, 11 studies have described the results of pelvic lymph node dissection in their cohort of patients. Results are summarized in Table 4.

### 3.7. Thromboprophylaxis and VTE Episodes

**Figure 2:**
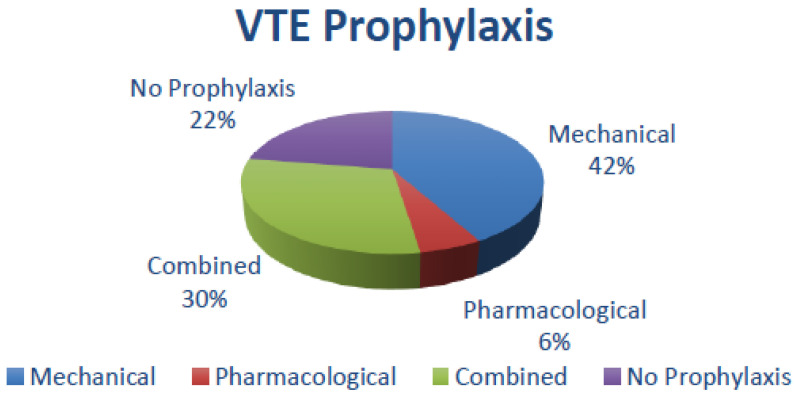
VTE Prophylaxis distribution in post-RP patients.

The results of different types of thromboprophylaxis, as well as the number of VTE episodes, are provided in Table 5. Fourteen studies have provided details about VTE prophylaxis (95,052 patients). Of these patients, 22.4% did not receive any form of VTE prophylaxis. Nearly half (41.8%) of patients received mechanical prophylaxis followed by 30% of patients receiving combined (mechanical, as well as pharmacological) prophylaxis, and lastly, 5.7% of patients received only pharmacological prophylaxis (Figure 2). 

### 3.8. Surgical Approaches and VTE Episodes

Total VTE episodes in open RP were discussed in 11 studies with a mean VTE incidence of 1.5%. Eight studies discussed VTE episodes in MIS having a mean incidence of 0.9%. Eight studies investigated DVT incidence in post-RP patients, accounting for 0.3%. Thirteen studies described PE incidence in post-RP patients and had a mean value of 1.5%. These results are tabulated in Table 6. 

### 3.9. Duration and Timing of Thromboprophylaxis

In most studies, mechanical prophylaxis was initiated in patients prior to surgery and continued till patient discharge from the hospital. In some studies, patients were also encouraged to ambulate on discharge [19]. 

However, there is a lack of uniformity in the case of PP in terms of both start and duration. Some studies started PP before surgery and continued every 8 hours till discharge from the hospital [17]. Others emphasized starting PP after surgery and continuing after for 4 weeks [18].

### 3.10. Statistical Results

a Statistical outcome of overall VTE occurrence

**Figure 3:**
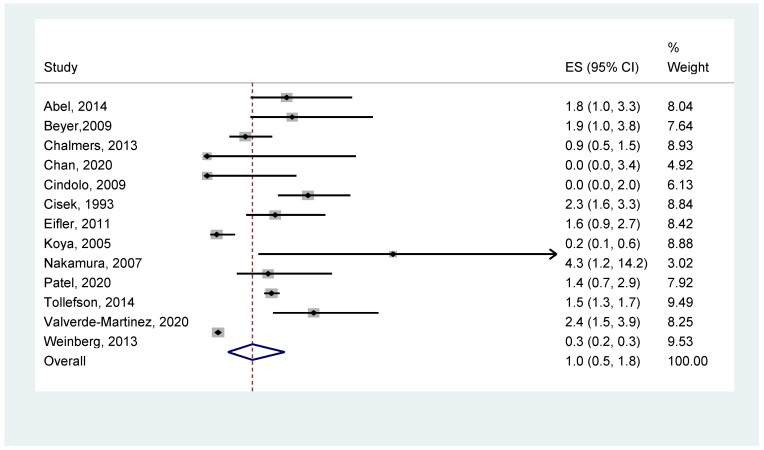
Overall VTE occurrence [14,17,18,19,20,21,22,25,26,28,29,30,31].

A summary of the meta-analysis results is summarized in Table 7. The first figures are the number of studies with valid data included in each analysis. Subsequently, details of the heterogeneity are reported, both in terms of the significance and the I^2^ values. The next figures are the pooled percentage of patients in which the outcome occurred for each method, presented with a corresponding confidence interval. The pooled results suggested that, for all patients combined, the occurrence of VTE was 1.0% (95% CI: 0.5% to 1.5%). The graphical illustration of the meta-analysis results is shown in Figure 3.

b Statistical outcome of VTE occurrence depending on the type of surgical procedure (Open/MIS) and whether PLND was performed or not.

A summary of the meta-analysis results is shown in Table 8. The first figures are the number of studies with valid data included in each analysis. Subsequently, details of the heterogeneity are reported in terms of the significance and the I^2^ values. The final columns give the differences in outcome between the two risk groups. The pooled difference in outcome between groups is expressed as a relative risk. The pooled figure is presented along with a corresponding confidence interval. The final column shows the *p*-values indicating the significance of the differences between risk groups. 

**Figure 4:**
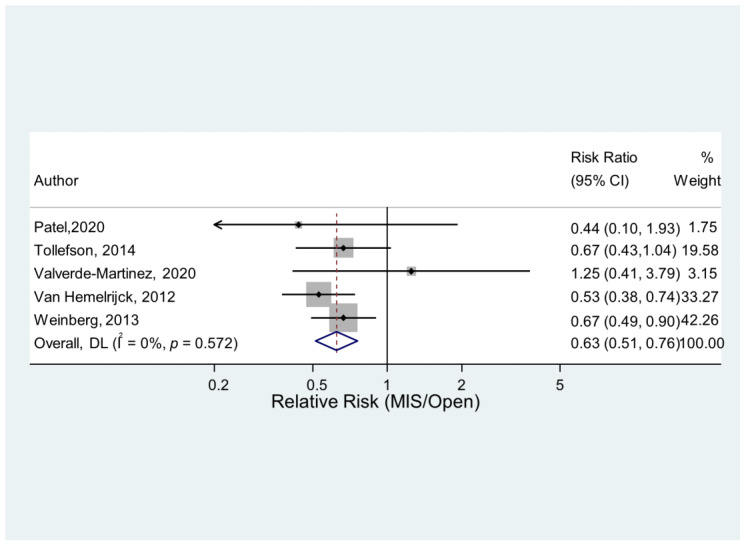
VTE in MIS and Open surgery [14,17,18,19,24].

The analysis suggested a statistically significant difference in outcome between MIS and Open procedures, based on data from five studies. The pooled results suggested a lesser occurrence of VTEs in MIS procedures compared to Open procedures. The number of VTEs was just over a third lower (0.63 times as large) in the MIS group when compared to the Open Group. There was little heterogeneity between the different studies. A graphical illustration of the meta-analysis results is shown in Figure 4.

**Figure 5:**
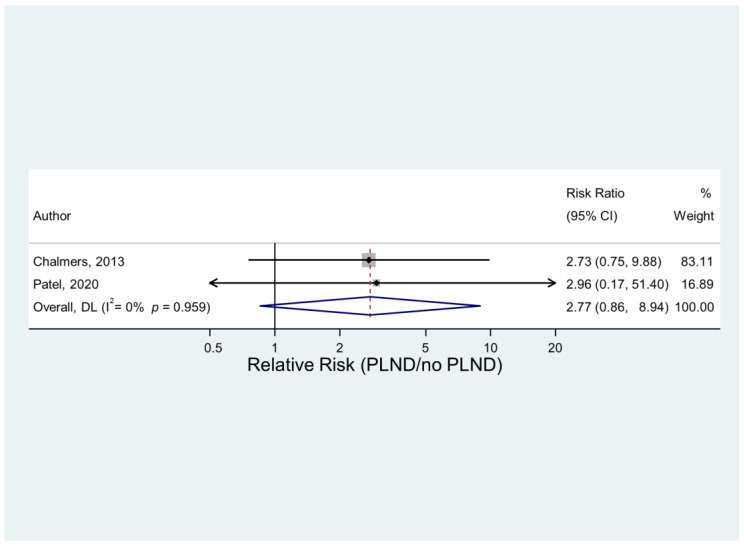
VTE in procedures with and without PLND [17,21].

There were two studies providing sufficient data to compare patients where PLND was used and not used. The data suggested some evidence that VTE was more common with the use of PLND. However, the result did not quite reach statistical significance. The pooled results suggested that a VTE was 2.8 times more likely when PLND was performed compared to when it was not. There was little heterogeneity between the two studies. A graphical illustration of the results is shown in Figure 5.

c Statistical outcome of VTE occurrence depending on the method of prophylaxis used (mechanical or combined).

The meta-analysis pooled together the results from studies with patients undergoing mechanical and combined prophylaxis methods. A summary of the meta-analysis results is shown in Table 9. The first figures are the number of studies with valid data included in each analysis. Subsequently, details of the heterogeneity are reported in terms of the significance and the I^2^ values. The next figures are the pooled percentage of patients with VTE for each method, presented with a corresponding confidence interval. The final column shows the *p*-values indicating the significance of the differences in VTE between groups. 

The pooled results suggested that 0.7% of patients underwent a VTE for the mechanical method, whilst 1.0% underwent VTE for the combined method. Statistically, no difference in VTE occurrence was found between the two methods. A graphical illustration of the results is shown in Figure 5.

## 4. Discussion

Venous thromboembolism (VTE) is a complex multifactorial clinical entity associated with significant morbidity and mortality and can present either as deep venous thrombosis (DVT) and/or concomitant pulmonary embolism (PE) [32]. VTE in malignancies is multifactorial—it can be cancer-related, as well as individual-related. A hypercoagulable state is induced directly by activating the blood clotting cascade and inducing pro-coagulant and inhibiting anticoagulant properties of endothelial cells, platelets, monocytes, and macrophages [33].

VTE does have a considerable impact on morbidity, mortality, and economic cost, and has led to the development of VTE risk stratification tools such as the Caprini risk assessment [32]. In addition to VTE risks associated with major surgical procedures and underlying malignancy, the American Heart Association (AHA) has identified additional factors including prior VTE, age, obesity, immobility, and family history. VTE risk stratification in surgical patients according to the Seventh American College of Chest Physicians Conference on Antithrombotic and Thrombolytic Therapy is provided in Supplementary Table S4.

Thromboprophylaxis, whether mechanical, pharmacological, or combination, is widely practiced across surgical specialties based on substantial evidence from randomized control trials (RCTs). However, there has been a lack of urology-specific evidence, leading to conflicting recommendations both nationally and internationally [13]. This becomes more complicated when individual urological procedures are considered for VTE prophylaxis. In the case of RP, the European Association of Urology (EAU) 2022 has provided guidelines for VTE prophylaxis based on available literature [34]. The EAU has considered a few factors including patient VTE risk factors (Low/Medium/High), type of surgical intervention (Open/laparoscopic/Robotic), extent of surgery (No PLND/Standard PLND/Extended PLND), and availability of evidence for it. The summarized results are tabulated in Table 10. The studies and research used for EAU guidelines are mainly non-urological, and rightly, they have admitted that the evidence base is limited for this guideline. On the other hand, the National Institute for Health, and Care Excellence (NICE), UK, recommends VTE Prophylaxis to people undergoing abdominal (gastrointestinal, gynecological, urological) surgery who are at increased risk of VTE. It recommends mechanical VTE prophylaxis on admission for people undergoing abdominal surgery, either anti-embolism stockings or intermittent pneumatic compression (until the patient is ambulatory). It also recommends pharmacological VTE prophylaxis for people undergoing abdominal surgery, including 7 days for non-cancer and 28 days (Extended) for major cancer surgery postoperatively [35]. The American Urological Association (AUA) recommends mechanical prophylaxis during MIS urological procedures, and pharmacological prophylaxis is reserved for high-risk patients only. For open urological procedures, the AUA recommends consideration of combined prophylaxis [36].

To our knowledge, this meta-analysis is the first of its kind specifically dealing with VTE prophylaxis in radical prostatectomy patients. We were able to establish a statistically significant higher incidence of VTE in open RP in comparison to MIS prostatectomy procedures. A similar observation has also been noticed in other abdominal procedures [37]. This analysis also revealed a higher incidence of VTE in patients undergoing RP, as well as PLND. A study in 2011 including 773 patients also demonstrated that there is a significant association between venous thromboembolism and radical prostatectomy plus pelvic lymph node dissection compared to radical prostatectomy only [25]. Lastly, we were able to establish that combined prophylaxis (mechanical and pharmacological) does not have an advantage over mechanical prophylaxis in RP. This implies that PP is not indicated for the routine MIS approach for RP procedures. This is a finding which has not been confidently proven before this meta-analysis.

Additionally, we did find that there are other well-established factors that can contribute to VTE occurrences in post-RP patients as well, including age, personal and family history of VTE. Some of the studies in this meta-analysis have discussed these contributing factors; however, the data were not enough to perform a statistical analysis. These have been summarized in Table 3. These factors are essential in stratifying high-risk cohorts of patients.

Lastly, we found that the duration of VTE prophylaxis after RP is quite variable across the globe. This is an issue that has been a bone of contention across surgical specialties both within and outside, and unfortunately, has been as such for more than a decade [38].

To summarize, this meta-analysis and systematic review revealed that VTE events in RP procedures were reduced by switching to MIS procedures in comparison to open procedures. Additionally, PLND increases the risk of VTE and should be considered a high-risk factor while stratifying patients for consideration of VTE prophylaxis. Lastly, PP in cases of RP does not have an added advantage to mechanical prophylaxis. This systematic review forms a basis and provides a platform for future, randomized controlled trials taking into account all variables for consideration of globally acceptable guidelines for VTE prophylaxis in RP surgeries. 

The limitations of this study include the scarcity of research available, the heterogenous nature of studies (the Newcastle–Ottawa scale might not have been appropriate to evaluate the quality of all studies), and the non-uniformity of VTE prophylaxis practices across the globe for RP. In the future, there is a need for a multicentric, randomized control trial to establish a VTE risk scoring system for RP patients and VTE prophylaxis guidelines to bring uniformity to this controversial practice.

## 5. Conclusions

This systematic review concludes that the overall incidence of VTE post-RP is 1%. The incidence of VTE in patients undergoing RP for prostate cancer is significantly less in minimally invasive (Laparoscopic/Robotic) procedures in comparison to open. Patients need to be stratified into risk groups using pre-existing guidelines and risk categories, while PLND should be considered an additional risk factor. Additional PP to mechanical methods may not be necessary in all cases and should be considered in high-risk patients only.

## Figures and Tables

**Table 1:** Newcastle–Ottawa Scale.

Author	Number of Awarded Stars in Each Domain		
	Selection	Comparability	Outcome
(1) Patel, H. D. [17]	****	**	**
(2) Valverde-Martinez, S. [18]	***	*	**
(3) Weinberg, A. [14]	***		**
(4) Tollefson, M. K. [19]	***		**
(5) Chan, S. [20]	**		**
(6) Chalmers, D. J. [21]	***		**
(7) Abel, E. [22]	***		**
(8) Dyer, J. [23]	**		*
(9) Van Hemelrijck, M. [24]	**		**
(10) Eifler, J. B. [25]	**		**
(11) Beyer, J. [26]	***		**
(12) Grasso, M. [27]	**		*
(13) Cindolo, L. [28]	**		*
(14) Nakamura, K. [29]	**		*
(15)Koya, M. [30]	***		*
(16) Cisek, L. [31]	**		*

* Each asterisk represents if an individual criterion within the subsection was fulfilled.

**Table 2:** Study characteristics.

Study	Study Type/ Time	Study Characteristics	Conclusion
(1) Patel, H. D. [17]	RCT (2017–2018)	A total of 501 patients enrolled, divided into two arms. Patients in the routine care (RC) arm received intermittent pneumatic compression devices without any pharmacological prophylaxis. (250 patients) Patients in the Pharmacological Prophylaxis (PP) arm received subcutaneous heparin (5000 units) given within 2 h prior to surgery and every 8 h after surgery until discharge from the hospital, as well as intermittent pneumatic compression devices. (251 patients)	This study concluded PP was not associated with a significant reduction in symptomatic VTE (0.8% vs. 2.0%), or a reduction in overall VTE (2.8% vs. 2.9%) when added to routine care with intermittent pneumatic compression devices and early ambulation. In subgroup analyses demonstrated that all 10 VTE events occurred in patients undergoing PLND. However, there was no statistically significant difference in the rate of symptomatic VTE among evaluated subgroups, including those evaluated by PLND status, Caprini score, Charlson Comorbidity Index, or surgical approach
(2) Valverde-Martinez, S. [18]	Retrospective (2013–2014)	Retrospective data of 610 men who underwent RP for organ-confined prostate cancer were evaluated. A total of 268 patients underwent ORP, 311 had LRP, and 31 had RALP. A total of 516 patients received Pharmacological or mechanical or combination while 94 received no prophylaxis. In this study, a VTE was defined as when the patient presented a symptomatic DVT or a PE.	In patients undergoing RP with or without risk factors of developing a thromboembolic event, a prophylactic treatment that combines early mobilization and pharmacological prophylaxis with LMWH would be indicated. Further, the use of PP reduces the risk of VTE in patients undergoing RP and this risk is not associated with the approach technique.
(3) Weinberg, A. [14]	Observational (2000–2010)	A total of 94,709 men who underwent RP were evaluated in this study. VTE prophylaxis was categorized as none, mechanical, pharmacologic, or combination The primary outcomes of interest were the use of VTE prophylaxis within 24 h of surgery and the occurrence of VTE.	This study concluded that the receipt of prophylaxis was associated with a reduction in the incidence of VTE by 33%. Further, this study found an overall low VTE rate among men undergoing RP, 0.25%, with no difference between surgical approaches.
(4) Tollefson, M. K. [19]	Retrospective (1987–2010)	This study reviewed the records of 18,472 consecutive patients, who underwent RP with PLND for the PCa. Venous thromboembolic events within 30 days of surgery were recorded.	Symptomatic VTE was found in 1.4% of patients in post-RP patients. This study concluded blood type, pelvic lymphadenectomy extent, and blood transfusion are significant risk factors for symptomatic VTE. Patients with non-O blood type were more likely to be diagnosed with VTE than patients with O blood. Conversely, patients with O blood type were more likely to experience bleeding requiring blood transfusion.
(5) Chan, S.Y. [20]	Prospective (2007–2010)	This was a prospective study on 109 Chinese patients who underwent RARP for PCa. Mechanical VTE prophylaxis was used in all patients followed by a postoperative duplex ultrasound scan on day 3. If this scan revealed the presence of DVT above the knee level, anticoagulation therapy was commenced immediately.	The post-RARP incidence of DVT in Chinese populations is not low. However, the majority of DVT cases are below knee level and asymptomatic. Older age and increased intra-operative blood loss were found to be associated with a higher risk of postoperative DVT. Smoking, a history of diabetes, BMI score, a longer operation time, lymph node dissection, and disease stage were found to have no influence on the risks of DVT in this study.
(6) Chalmers, D. J. [21]	Prospective (2007–2011)	This study compared the rates of postoperative VTE in a group of patients undergoing RP with perioperative heparin prophylaxis versus those without heparin prophylaxis. All patients underwent RARP and had mechanical VTE prophylaxis.	The risk of VTE in patients undergoing RP is low and not significantly reduced with the administration of prophylactic heparin/SCDs compared with SCD alone. Heparin prophylaxis for patients undergoing RALP already receiving SCDs may be unnecessary for the average-risk patient population. BMI, blood loss, duration of surgery, and performance of lymph node dissection were not associated with VTE.
(7) Abel, E. [22]	Retrospective (2007–2011)	A total of 549 consecutive patients having RARP were identified, with a median follow-up period of 8 months. The purpose of this study was to evaluate the effect of operative time on the incidence of symptomatic VTEs in patients undergoing RARP.	This study found that an increase in operative time, high BMI, and the need for transfusion increased VTE risk. Heparin prophylaxis was not associated with a significant VTE risk reduction, but was also not associated with a significant increase in estimated blood loss or transfusion rate.
(8) Dyer, J. [23]	Retrospective (2009–2010)	NHS UK, HES database data were obtained for all patients undergoing common urological procedures in NHS trusts throughout England between April 2009 and April 2010 This study aimed to elucidate the rate of procedure-specific postoperative VTE in patients undergoing a range of urological surgery	This study revealed that there is the potential benefit of prolonging the use of thromboprophylaxis in high-risk patients. There is an apparent lack of need for routine thromboprophylaxis in patients undergoing low-risk procedures.
(9) Van Hemelrijck, M. [24]	Retrospective (2002–2010)	This study used nationwide population-based cohort data based on the National Prostate Cancer Register (NPCR) of Sweden to assess in detail the risk of VTE associated with different urological surgical treatments. Data from 45,065 men were analyzed.	This study concluded that a large proportion of thromboembolic events attributable to surgery occurred during days 14–28 after PCa surgery. Prophylactic measures, both pharmacologic and physiotherapeutic, should be used after all major surgery for PCa and seem particularly important for men undergoing PLND, as well as for men with a previous history of VTE.
(10) Eifler, J. B. [25]	Retrospective (2001–2009)	A total of 770 consecutive patients were included and underwent LRP with or without PLND. VTE was twice as likely in patients treated with RP who underwent PLND as in those who did not.	PLND during radical prostatectomy increases the risk of VTE. Patients in whom VTE developed had no statistically significantly increased operative time or the number of lymph nodes removed compared to those in whom VTE did not develop.
(11) Beyer, J. [26]	Prospective (2001–2003)	A cohort of 411 patients undergoing standardized RP with PLND was prospectively evaluated regarding risk factors for and incidence of VTE. VTE prophylaxis was performed with graduated compression stockings, early mobilization, and a daily standard dose of low molecular weight from the day before surgery until discharge.	VTE is common after radical prostatectomy. A significant number of patients develop VTE usually after day 8. Most of the VTE events in this study were asymptomatic and were limited to calf veins.
(12) Grasso, M. [27]	Retrospective (1999–2006)	In this study, 500 ORP patients were evaluated for clinical signs, laboratory parameters, auto-transfusions, age, PSA, Hb levels, and the number of transfusions.	This study suggests multiple factors can help in the prevention of VTE in RP including, LMWH starting within 24 h, associated with preoperative blood donation, intra-operative haemodilution, compression stockings, surgical care to avoid lymphocele, and early mobilization.
(13) Cindolo, L. [28]	Prospective (2004–2006)	A total of 184 consecutive ORP patients received VTE prophylaxis with enoxaparin and IPC of the thigh. Patients were provided with a questionnaire to evaluate the comfort and tolerability of a compression device. The patients were monitored for complications and development of VTE for up to 4 weeks postoperatively.	This study concluded that external pneumatic compression associated with LMWH can be easily provided with acceptable compliance and safety, justifying routine use after RP.
(14) Nakamura, K. [29]	Prospective (2003–2005)	A total of 47 patients undergoing ORP were evaluated for the development of VTE episodes using combined prophylaxis of PCS, and initiation of enoxaparin in the immediate postoperative period.	They concluded enoxaparin may be useful in preventing VTE, in adjunct with PCS, in patients undergoing ORP.
(15) Koya, M. [30]	Prospective (1992–2004)	A total of 1373 patients undergoing ORP were evaluated for the development of VTE episodes using mechanical devices.	This study concludes that due to mechanical devices and early mobilization, and the potential risk and expense of heparinoid prophylaxis, the routine use of heparinoid prophylaxis is questionable.
(16) Cisek, L. [31]	Prospective (1982–1993)	In this study, 1330 ORP patients were evaluated for the role of SCD in the prevention of VTE.	This study could not demonstrate a beneficial effect of SCD on postoperative thromboembolic complications.

BMI—Body Mass Index; DVT—Deep Vein Thrombosis; HES—Hospital Episode Statistics; LMWH—Low Molecular Weight heparin; IPC—Intermittent pneumatic compression; ORP—Open Radical Prostatectomy; PCa—Prostate Cancer; PCS—Pneumatic Compression Stocking; PLND—Pelvic Lymph Node dissection; PP—Pharmacological Prophylaxis; RARP—Robot-assisted radical prostatectomy; SCD—Sequential Compression Device; VTE—Venous thromboembolism.

**Table 3:** Demographics and VTE risk factors.

Study	Total Patients	Mean Age	Mean BMI kg/m^2^	Family History (%)	VTE Background (%)	Smoking	Overall Risk Assessment (in %)			Caprini Score	Remarks in Relation to VTE Risk Factors/Scores
							Low	Int	High		
(1) Patel, H. D. [17]	501	62	27.4	-	-	-	-	-	-	6	The study concluded that most patients with prostate cancer undergoing RP are relatively healthy. Our study suggests that PP may be deferred based on surgeon preference up to a Caprini score of 7; PP may be justified for higher-risk patients with scores of 8.
(2) Valverde-Martinez, S. [18]	610	64.1	28.03	-	-	-	94.8	4.1	1.1	-	This study concluded that with respect to the PP used in different thromboembolic risk groups, there were differences in the low-risk group, but not in the intermediate and high-risk groups; this was probably due to the fact that this group covered 95% of the cases in the series.
(3) Weinberg, A. [14]	94,709	-	-	-	-	-	-	-	-	-	-
(4) Tollefson, M. K. [19]	18,472	63	27.7	-	-	-	-	-	-	-	They concluded that patients with VTE were significantly older than those not diagnosed with VTE (median age 65 vs. 63 years, *p* < 0.001).
(5) Chan, S.Y. [20]	109	65.7	<23 (33) >23 (67)	-	-	18.1	-	-	-	-	This study concluded that there was no difference in the incidence of DVT between patients with a history of smoking or diabetes or a high body mass (BMI) index and those without.
(6) Chalmers, D. J. [21]	1486	59.9	28.1	-	-	-	-	-	-	-	In this study, BMI was not found to be associated with VTE.
(7) Abel, E. [22]	549	59.8	-	-	1.6	43.8				-	A 5-point increase in body mass index was associated with an increased risk of VTEs (odds ratios of 2.0).
(8)Dyer, J. [23]	3213	72.5	-	-	-	-	-	-	-	-	-
(9) Van Hemelrijck, M. [24]	16,304	-	-	-	0.6	-	-	-	-	-	A previous history of VTE is a risk factor in patients undergoing RP.
(10)Eifler, J. B. [25]	773	57.8	27.3	-	-	-	-	-	-	-	A high incidence of VTE was found in patients with BMI in the top quartile who concomitantly underwent PLND.
(11) Beyer, J. [26]	411	65.0	27.0	4.0	4.8		-	-	-	-	A statistically higher risk was found in patients with a personal history of VTE; however, family history was not found with increased risk.
(12) Grasso, M. [27]	500	65.0	-	-	-	-	-	-	-	-	-
(13) Cindolo, L. [28]	184	69.0	>25 (30%)	-	-	28	-	-	-	-	-
(14) Nakamura, K. [29]	47	64.0	-	-	-	-	-	-	-	-	-
(15)Koya, M. [30]	1364	60.8	-	-	-	-	-	-	-	-	-
(16) Cisek, L. [31]	1300	-	-	-	-	-	-	-	-	-	-

**Table 4:** Details of surgical procedures.

Study	Total Procedures	Open	Laparoscopic	Robotic	Unknown	PLND (%)
(1) Patel, H. D. [17]	501	124	-	377	-	83.5 (419)
(2) Valverde-Martinez, S. [18]	610	268	311	31	-	-
(3) Weinberg, A. [14]	94,709	68,244	-	26,465	-	-
(4) Tollefson, M. K. [19]	18,472	16,374	-	2098	-	100
(5) Chan, S. [20]	109	-	-	109	-	33.94 (37)
(6) Chalmers, D. J. [21]	1486	-	-	1486	-	55
(7) Abel, E. [22]	549	-	-	549	-	12.9 (71/549)
(8) Dyer, J. [23]	3213	-	-	-	3213	-
(9) Van Hemelrijck, M. [24]	16,304	11,137	-	5167	-	21.6 (3258/16,304)
(10) Eifler, J. B. [25]	770	-	770	-	-	60.8 (468/770)
(11) Beyer, J. [26]	411	411	-	-	-	100
(12) Grasso, M. [27]	500	500	-	-	-	-
(13) Cindolo, L. [28]	184	184	-	-	-	100
(14) Nakamura, K. [29]	47	47	-	-	-	87 (41/47)
(15)Koya, M. [30]	1373	1373	-	-	-	67 (920/1373)
(16) Cisek, L. [31]	1300	1300	-	-	-	-
Total	140,541	100,088 (71.21%)	1084 (0.77%)	36,156 (25.72%)	3213 (2.28%)	33.82% (6229/18,417)

**Table 5:** Thromboprophylaxis and VTE episodes.

	Thromboprophylaxis				VTE Symptomatic Episodes (in %)			
	N	M	P	C	N	M	P	C
(1) Patel, H. D. [17]	-	250	-	251	-	2.0	-	0.8
(2) Valverde-Martinez, S. [18]	94	25	516	21	2.5			
(3) Weinberg, A. [14]	20,438	35,591	4945	7720	0.25			
(4) Tollefson, M. K. [19]	-	-	-	18,472	1.47			
(5) Chan, S. [20]	-	109	-	-	0.09			
(6) Chalmers, D. J. [21]	-	564	-	922	-	1.0	-	0.7
(7) Abel, E. [22]	-	540	-	9	1.8			
(8) Dyer, J. [23]	-	-	-	-	1.0			
(9) Van Hemelrijck, M. [24]	-	-	-	-	1.2			
(10) Eifler, J. B. [25]	-	770	-	-	1.5			
(11) Beyer, J. [26]	-	-	-	411	1.9			
(12) Grasso, M. [27]	-	-	-	500	0.2			
(13) Cindolo, L. [28]	-	-	-	184	0			
(14) Nakamura, K. [29]	-	-	-	47	4			
(15)Koya, M. [30]	-	1373	-	-	0.21			
(16) Cisek, L. [31]	784	516	-	-	2.3			

**Table 6:** Surgical approach and VTE incidence.

Study	VTE Incidence Procedure Specific		DVT Incidence (in %)		PE Incidence (in %)		PLND (VTE)	Post-Op Bleeding Episodes (in %)
	O	MIS	O	MIS	O	MIS		
(1) Patel, H. D. [17]	2.4	1.1	-		-		1.7	1.1
(2) Valverde-Martinez, S. [18]	2.5		-		1.4		-	-
(3) Weinberg, A. [14]	0.3	0.2	-		0.1	0.1	-	-
(4) Tollefson, M. K. [19]	1.5	1.0	-		1.8		1.47	-
(5) Chan, S.Y [20]	-	0.9	-		0.0		-	-
(6) Chalmers, D. J. [21]	-	0.9	-		-		1.2	-
(7) Abel, E. [22]	-	1.8	-		-	0.5	-	-
(8) Dyer, J. [23]	1.0		-		-		-	-
(9) Van Hemelrijck, M. [24]	1.5	0.8	0.9	0.6	0.6	0.2	-	-
(10)Eifler, J. B. [25]	-	-	0		1.5		-	-
(11) Beyer, J. [26]	1.9	-	0.9		0.9		-	-
(12) Grasso, M. [27]	0.2	-	0		0.2		-	-
(13) Cindolo, L. [28]	0	-	0		0		-	-
(14) Nakamura, K. [29]	4	-	0		4		-	2.1
(15)Koya, M. [30]	0.21	-	0.21		0		-	-
(16) Cisek, L. [31]	2.3	-	0.45		1.3		-	-

DVT—Deep Vein Thrombosis; MIS—Minimally Invasive Surgery; O—open surgery; PE—Pulmonary Embolism; PLND—Pelvic Lymph Node Dissection; VTE—Venous Thrombo-embolism.

**Table 7:** Summary of meta-analysis results for overall VTE occurrence.

Outcome	Method	Number	Heterogeneity		Outcome Occurrence
		Studies	*p*-Value	I^2^	% (95% CI)
VTE	All combined	16	<0.001	97%	1.0 (0.5, 1.5)

**Table 8:** Summary of meta-analysis results for VTE occurrence based on approach and PLND.

Comparison	Number	Heterogeneity		Group Difference	
	Studies	*p*-Value	I^2^	RR (95% CI) (*)	*p*-Value
MIS/Open	5	0.55	0%	0.63 (0.52, 0.77)	<0.001
PLND/no PLND	2	0.96	0%	2.79 (0.86, 8.94)	0.09

(*) Relative risks expressed as per the ‘Comparison’ column.

**Table 9:** Summary of meta-analysis results for VTE occurrence by Prophylaxis method.

Prophylaxis	Number	Heterogeneity		VTE Occurrence	Method Diff.
Method	Studies	*p*-Value	I^2^	% (95% CI)	*p*-Value
Mechanical	5	0.002	76%	0.7 (0.1, 1.6)	0.42
Combined	6	0.07	51%	1.0 (0.5, 1.6)	

**Table 10:** EAU guidelines for VTE prophylaxis.

**Open Radical Prostatectomy (+/− PLND)**			
Pharmacological^#^	Low Risk	Suggests	weak, moderate-quality evidence
	Medium/High Risk	Recommends	strong, moderate- or high-quality evidence
Mechanical*	All patients	Suggested	weak, low-quality evidence
**Open radical prostatectomy with extended PLND**			
Pharmacological^#^	All patients	Recommends	strong, moderate, or high-quality evidence
Mechanical*	All patients	Suggests	weak, low-quality evidence
**Laparoscopic Radical prostatectomy (Without PLND)**			
Pharmacological^#^	Low Risk	Recommends (Against)	strong, moderate-quality evidence
	Medium and high risk	Suggests (Against)	weak, moderate- or high-quality evidence
Mechanical*	Low risk	Suggests (Against)	weak, low-quality evidence
	Medium and high risk	Suggests	weak, low-quality evidence
**Laparoscopic Radical prostatectomy (With Standard PLND)**			
Pharmacological^#^	Low Risk	Recommends (Against)	strong, moderate-quality evidence
	Medium Risk	Suggests (Against)	weak, moderate- or high-quality evidence
	High Risk	Recommends	strong, high-quality evidence
Mechanical*	All patients	Suggests	weak, low-quality evidence
**Laparoscopic Radical prostatectomy (With Extended PLND)**			
Pharmacological^#^	Low Risk	Suggests (Against)	weak, moderate-quality evidence
	Medium Risk	Suggests	weak, high-quality evidence
	High Risk	Recommends	strong, high-quality evidence
Mechanical*	All patients	Suggested	weak, low-quality evidence
**Robotic Radical prostatectomy (Without PLND)**			
Pharmacological^#^	Low Risk	Recommends (Against)	strong, moderate-quality evidence
	Medium and High Risk	Suggests (Against)	weak, moderate-quality evidence
Mechanical*	Low Risk	Suggests (Against)	weak, low-quality evidence
	Medium and High Risk	Suggests	weak, low-quality evidence
**Robotic Radical prostatectomy (With Standard PLND)**			
Pharmacological^#^	Low Risk	Recommends (Against)	strong, moderate-quality evidence
	Medium Risk	Suggests	weak, moderate-quality evidence
	High Risk	Suggests	weak, moderate-quality evidence
Mechanical*	All patients	Suggests	weak, low-quality evidence
**Robotic Radical prostatectomy (With Extended PLND)**			
Pharmacological^#^	Low Risk	Suggests (Against)	weak, moderate-quality evidence
	Medium Risk	Suggests	weak, moderate-quality evidence
	High Risk	Recommends	strong, moderate-quality evidence
Mechanical*	All patients	Suggests	weak, low-quality evidence

Pharmacological^#^—for 4 weeks post-operatively; Mechanical*—until ambulation.

## Data Availability

Data is contained within the article or supplementary material.

## Supplementary Materials

The following supporting information can be downloaded at: https://www.mdpi.com/article/10.3390/jcm12123979/s1, Table S1: PRISMA Checklist; Table S2: Search Strategy; Table S3: Clinicopathological Characteristics; Table S4: Venous Thromboembolism (VTE) Risk Stratification in Surgical Patients.
